# Comparison of Students With and Without Problematic Smartphone Use in Light of Attachment Style

**DOI:** 10.3389/fpsyt.2019.00681

**Published:** 2019-09-18

**Authors:** Christiane Eichenberg, Markus Schott, Athina Schroiff

**Affiliations:** ^1^Institut für Psychosomatik, Medizinische Fakultät, Sigmund Freud PrivatUniversität Wien, Vienna, Austria; ^2^Fakultät für Medizin, Technische Universität München, Munich, Germany

**Keywords:** smartphone, Internet, addiction, attachment style, online

## Abstract

**Background:** Nowadays, media addictions are especially of high relevance to psychotherapeutic practice. More recently, this particularly includes excessive smartphone usage. Even though a growing number of scientific literature and also mainstream media highlight problematic smartphone use as a serious health problem, there is only little research on this issue.

**Objective:** The aim of this study was to examine this phenomenon with a focus on attachment-specific differences between students with and without problematic smartphone use.

**Method:** A survey was carried out on all enrolled students of the Sigmund Freud University Vienna. The Smartphone Addiction Scale (SPAS) was used to differentiate between students with and without problematic smartphone use. The attachment style was assessed using the Bielefeld Partnership Expectations Questionnaire (BFPE).

**Results:** Of the total sample, 75 of the students (15.1%) showed a problematic smartphone use. A positive correlation between excessive smartphone usage and an insecure attachment style was found.

**Discussion:** Therapy for problematic smartphone use should be carried out in light of patient’s attachment style. Further research into other factors of mental stress and personality is needed to better understand problematic smartphone use.

## Introduction

We spend more time with our smartphone than with any other human. Nearly everyone has their mobile device either directly on the body or at least in close vicinity around-the-clock. Teenagers between the age of 18 and 24 years look at their smartphone an average of 214 times a day (1). Not only do people use their smartphone more and more frequently but also (almost) everywhere: at work, at the home on the couch, while shopping, during the commute in buses and trains, and at lunch or dinner. Smartphone use is regulated by law for drivers, but not for pedestrians in traffic.

So far, smartphone addiction has no independent diagnosis in the current classification systems for mental disorders, *International Classification of Diseases*, 10th revision (ICD-10) (2), and *Diagnostic and Statistical Manual of Mental Disorders*, 5th Edition (DSM-5) (3); and, furthermore, it is a controversial term in this field of research (4). Therefore, in this study, the more neutral term “problematic smartphone use” will be applied. According to Biang and Leung (5), characteristics of a problematic smartphone use are similar to the diagnostic criteria of the more researched Internet addiction. Therefore, as the smartphone can be seen as a medium that offers many possibilities and functions to access the Internet, theoretical models for excessive media use and Internet addiction can be transferred to problematic smartphone use (6). Still, even this diagnosis is problematic as behavioral addictions can only be found to a very limited extent in the ICD-10 and DSM-IV-TR (7). The core diagnostic characteristics of Internet addiction, although not yet uniformly defined, consist of the following: mental preoccupation with the Internet; development of tolerance; social withdrawal; frustrations with relapse; withdrawal symptoms (irritability, anxiety, and sadness); loss of interest in previous hobbies or activities; continuation of excessive consumption despite the knowledge of the resulting psychosocial problems; dysfunctional affect regulation; lying to friends, family members, or therapists to conceal actual consumption; and the loss of a meaningful relationship, job, or apprenticeship or career opportunities (8–11).

Similar diagnostic criteria for problematic smartphone use are proposed: compulsive behavior, functional impairment, withdrawal, and tolerance (12). Surveys report problematic smartphone use in 24% to 50% of the respondents (13). Prevalence of problematic smartphone use among students is as high as 24.8% to 27.8% and is steadily increasing every year (14). There are adverse effects on physical and mental health due to problematic smartphone use. Negative physical effects include neck pain (15), eye problems, and muscular pain (16). Regarding mental health, recent studies showed a connection between increased smartphone use and sleep disturbances (17) and problems with interpersonal relationships (18). Unfortunately, little is known about etiopathogenetic factors contributing to problematic smartphone use. Smartphones offer a wide variety of additional possibilities and functions that even further amplify the likelihood to develop obsessive behaviors (19). In this context, Autenrieth (20) emphasized the constant availability of the Internet. In particular, social networks, such as Facebook, Instagram, and Snapchat, play a major role in increasing the addictive potential. Roberts et al. (21) found that females reported spending significantly more time with their smartphones than males, and that particularly texting, sending e-mails, and using social media were the most time-consuming activities. In another study, Smetaniuk (22) reported that lower age, depression, and extraversion were correlated with higher scores on measures of problematic smartphone use. A Korean study (23) demonstrated that individuals with lower education levels were more likely to be diagnosed with problematic smartphone use.

Attachment theory offers a possible model to explain the development of problematic smartphone use. Attachment theory is based on the work of John Bowlby and Mary Ainsworth (24). The attachment system can be understood as a biologically and evolutionarily anchored motivational and behavioral system, which is mediated through interaction with attachment figures and in turn influences affect regulation, relationships, and their neurobiological correlates. According to Bartholomew and Horowitz (25), there are one “secure” and three “insecure” attachment styles: “avoidant-closed,” “ambivalent-clingy,” and “ambivalent-closed.” Attachment style as a key feature in explaining various psychopathologies in the context of affective and interpersonal problems has been well documented (26, 27). Schuhler et al. (28) emphasized the connection between insecure attachment styles, long-lasting crises and conflicts in close relationships, anxiety in a social context, and Internet addiction. Schuhler (29) highlighted that the Internet offers a virtual world of relationships in which problematic real-world attachment experiences can be compensated. This assumption is similar to Brisch’s (30) model of the reference object as a key component to addictions. Problematic smartphone use in contrast to other addiction disorders, such as the gambling addiction, not only replaces negative feelings by intoxication but also offers a replacement for a lack in secure social relationship due to insecure attachment styles (30). Smartphones offer numerous opportunities to communicate and establish relationships through social networks as well as a more easy way to manage one’s self-presentation. This is a particularly important factor for the smartphones’ addictive potential, considering that insecure attachment styles often accompany a disturbed self-perception (31). Even if the smartphone is used for other reasons, for example, as a place to retreat, it still offers numerous opportunities to engage in social relationships than do other non-substance and substance addictions. In summary, excessive smartphone use can be understood as a dysfunctional attempt to compensate for deficits in social relationship due to insecure attachment styles (32, 33). An association between attachment style and addiction was confirmed by Eichenberg et al. (34), Unterrainer et al. (35), and Hiebler-Ragger et al. (36). In light of the discussed research, the purpose of this study was to investigate whether insecure attachment styles are also positively associated with problematic smartphone use.

## Method

### Study Design

An online survey was carried out on all active students currently enrolled at Sigmund Freud University Vienna (*N* = 1,836). Students were contacted to participate through mail. Research data were collected with Unipark, a web-based survey software. A pretest was carried out with nine participants. Returns were analyzed, and the instrument was revised regarding its practicability, comprehensibility, and completeness of item formulation. The survey was online and available between 17 March 2017 and 13 May 2017. Before participants could start the questionnaire, information about study design and confidentiality was provided. The survey was viewed 843 times during the survey period. Most participants (23%) quit the questionnaire at the first page. Only about 8% of participants did not continue the questionnaire after the second page. Therefore, the overall dropout rate of 40.04% is acceptable (37). In total, there were 497 completed records. Completing the questionnaire took about 15 min. Ethical approval was obtained from the Sigmund Freud University Vienna ethics committee.

### Material

Data on age, gender, nationality of participants, and field of study were collected. In addition, participants were asked to indicate how much they used their smartphone and for what services. Participants could choose between four categories: information search, utilities (make photos/videos, e-mail, and dictionary), entertainment (games, listening to music, and e-book), and socializing/communication (sms and calls). Each category was rated on a 5-point Likert scale ranging from “never” to “daily.” Subsequently, the following questionnaires were collected in the following order.

#### Smartphone Addiction Scale (SPAS)

The Smartphone Addiction Scale (SPAS) (5) was used to assess symptoms of problematic smartphone use. This instrument assesses five primary symptoms: ignoring harmful consequences, excessive thinking about using the smartphone, inability to control desire, loss of productivity, and anxiety (5). The questionnaire consists of 19 items and three inventories: Mobile Phone Problematic Use Scale (MPPUS), Internet Addiction Test, and the Television Addiction Scale. The authors report a reliability coefficient of 0.70.

For this study, only a differentiation between participants with and without problematic smartphone use was needed. Therefore, only the eight items directly assessing problematic smartphone use were used. Items assessing Internet or television addiction were not used in the current study. For data analysis, the five-point Likert scale was dichotomized. Answers were summed up, resulting in overall values between 0 and 8. Subjects with a score of 5 or more were diagnosed with a problematic smartphone use.

#### Bielefeld Partnership Expectations Questionnaire (BFPE)

The Bielefeld Partnership Expectations Questionnaire (BFPE) was used to assess the attachment style of participants. This inventory consists of 31 items that are rated on a 5-point Likert scale ranging from 0 (“completely disagree”) to 4 (“completely agree”). The questionnaire assesses five attachment styles. While the “secure” attachment style (25) is divided into two further categories (“secure” and “conditionally secure”), the remaining three (“avoidant-closed,” “ambivalent-clingy,” and “ambivalent-closed”) are equivalent to the ones originally described. The reliability of the scales (Cronbach alpha = .77 to.89) is satisfactory.

The Bielefeld questionnaire is different from the others in two ways: (1) attachment style is operationalized as configurations of scale scores, which allow qualitative distinctions in terms of functioning, and (2) five empirically identified attachment styles are distinguished. Nonetheless, validation of the classifications with a German translation of the “Adult Attachment Scale” (AAS) yielded good results (38).

### Statistical Analysis

The Statistical Package for the Social Sciences Program (SPSS Version 24) was used for data input, processing, and statistical analyses. Based on the data obtained with the BFPE (38), a cluster analysis was performed. Accordingly, the individuals were allocated to the five attachment styles “secure,” “conditionally secure,” “ambivalent-clingy,” “ambivalent-closed,” and “avoidant-closed.” Subsequently, the five attachment styles were dichotomized into the variables “safe” and “insecure” attachment styles. Finally, using the chi-square test, attachment style and smartphone use were tested for significant differences.

## Results

### Sample

The total sample of *N* = 497 consisted of *n* = 120 men (24.2%) and *n* = 377 women (75.8%). The majority of the surveyed subjects (72.8%) were from Germany, 13.6% from Austria, and only 3% from other countries. Some respondents (10.6%) did not report their nationality. Participants were between 17 and 70 years old, with average age being *M* = 19.38 years (*SD* = 16.50). Most participants studied either psychotherapy (*n* = 286, 57.5%) or psychology (*n* = 125, 25.2%). Only 16.5% studied medicine (*n* = 82) and 0.6% law (*n* = 4). This distribution was expected considering the composition of active students at the Sigmund Freud University.

### Smartphone Use

For *n* = 19 subjects (1.4%), essential data were missing for a comprehensive analysis. According to the criteria and the cutoff of the SPAS, *n* = 75 (15.1%) participants were diagnosed with a problematic smartphone use. Of these participants, 86.7% were female and only 13.3% male. However, this gender ratio is in line with the gender distribution of the total sample.

### Smartphone Services

All presented services were used approximately to the same extent. The most commonly used smartphone service was “communication” (*M* = 4.9, *SD* = .5). The least used service was “entertainment” (*M* = 4.4, *SD* = 1.02). Participants used their smartphone for information research and other utilities equally often (*M* = 4.6, *SD* = .77).

### Attachment Style

About one third of the total sample (37%) had an “ambivalent-clingy” attachment style; 41% had an “ambivalent-closed” attachment style. Only 8.7% of the subjects showed a “secure” attachment style; similarly, only few participants could be classified as “conditionally secure” (5.6%) or “avoidance-closed” (7.6%) attached. These results are not consistent with the distribution reported by Höger and Buschkämper (38). Therefore, there is no balance between safe and insecure attached subjects. To ease statistical analyses and to create more balanced groups, the five attachment styles were dichotomized into “secure attachment style” and “insecure attachment style.”

### Smartphone Use and Attachment Style

Interference statistical analysis of the data showed that students with a problematic smartphone use differed significantly from students without a problematic smartphone use in their attachment style (χ^2^(1) = 7.43; *p* = .006) (see **Table 1**). Students with a problematic smartphone use (*n* = 75) mostly had an “insecure” attachment style (*n* = 72), and only few (*n* = 3) had a “secure” attachment style. Considering individuals without a problematic smartphone use (*n* = 415), it can be seen that more subjects (*n* = 66) than expected (*n* = 58.4) showed a secure attachment style and less than expected an insecure attachment style.

**Table 1:** Problematic smartphone use and dichotomized attachment style.

		Insecure	Secure
Unproblematic smartphone use	*n* Expected	349 356.6	66 58.4
Problematic smartphone use	*n* Expected	72 64.4	3 10.6
Total	*n* Expected	421 421	69 69

Accordingly, significantly more than expected students with a problematic smartphone use had an insecure attachment style and significantly more students without a problematic smartphone use a secure attachment style. The contingency coefficient of *C* = .12 indicates a weak effect.

As expected, significant differences were also found with respect to individual attachment styles (*C* = .18, Ckorr = .22, χ^2^(4) = 16.31, *p* = .003) (see **Table 2**). Findings show that participants with a problematic smartphone use had a significant higher likelihood to have an “ambivalent-closed” attachment style (*K* = 2.3). In addition, there were significantly less participants with a problematic smartphone use that had a “conditionally secure” attachment style (*z* = −2.0).

**Table 2:** Problematic smartphone use and individual attachment styles.

		Avoidant-closed	Conditionally secure	Secure	Ambivalent-clingy	Ambivalent-closed
Unproblematic smartphone use	*n* Expected	35 31.3	27 22.9	39 35.6	155 153.3	159 171.9
Problematic smartphone use	*n* Expected	2 5.7	0 4.1	3 6.4	26 27.7	44 31.1
Total	*n* Expected	37 37.0	27 27.0	42 42.0	181 181.0	203 203.0

In summary, according to the available data, the “ambivalent-close” attachment style is associated with excessive smartphone use. Out of 75 subjects classified with a problematic smartphone use, a large majority (*n* = 70, 93.3%) were located in this attachment category.

## Discussion

Even though a growing number of scientific literature and also mainstream media highlight problematic smartphone use as a serious health problem, only little research investigates possible etiopathogenetic factors. Since an association between attachment style and substance dependence has been widely demonstrated (29, 34), the aim of the present study was to investigate how people differ in their attachment style in regard to their tendency to problematically use smartphone. In this study, 15.1% of the participants showed problematic smartphone use. This result is comparable with prevalence rates reported in the available literature (39, 40).

A small part of the random sample (8.7%) showed a “secure” attachment style; similar numbers of participants could be classified as “conditionally secure” (5.6%) or “avoidance-closed” (7.6%). One third of the total sample (37%) showed an “ambivalent-clingy” attachment style and 41% an “ambivalent-closed” attachment style. These findings are not consistent with the distribution of attachment styles reported by Höger and Buschkämper (38). It has been reported in several other studies that the proportion of students with a secure attachment style is decreasing recently (41). There are various explanations for this difference, especially with regard to older prevalence numbers. For example, recent economic uncertainties can have an influence on interpersonal development. Furthermore, changes and particularly an increase in media use can influence the development of participants’ attachment styles (41). Overall, the assumption that insecure people more often show an increased tendency to problematically use smartphones was confirmed; “ambivalent-closed” attachment styles were especially associated with a problematic smartphone use. Similar results are reported in a study by Eichenberg et al. (34), which found a significant relationship between Internet addiction and an insecure attachment style. As individuals with an “ambivalent-closed” attachment style particularly demonstrate difficulties with social acceptance and opening up to others and simultaneously show a distinct desire to connect with others, it can be assumed that in particular the social compensatory component plays a significant role in the context of excessive smartphone use. Individuals with an “ambivalent-closed” attachment style use the smartphone to compensate for their “real” deficits regarding interpersonal relationships. The anonymity of the Internet allows to create a new representation of the self, which could allow these individuals to compensate for dreaded “real” acceptance problems.

Based on the finding that primary attachment styles in individuals differ depending on substance abused, Schindler et al. (42) argued that an attempt may possibly be made to compensate for specific attachment deficits by using different substances. On the other hand, Eichenberg et al. (34) showed that attachment styles do not explain differences in regard to online services used. Accordingly, the question arises as to whether primarily the medium or the content has an influence on the addictive potential and how this relates to the attachment style. Future research into smartphone or Internet addiction needs to consider the context of different services used. In light of the discussion on etiopathogenetic factors of problematic smartphone use in various areas as well as the influence of media on the style of attachment, long-term research of users with a problematic smartphone use is needed. As a result, it can be concluded whether the attachment style can act as a disposition and thus favors such a development. Furthermore, it is necessary to identify additional risk factors that favor the development of a problematic smartphone use. For example, various studies underline that excessive media consumption is associated with certain personality traits. In particular, Love and Kewly (43) found that extroversion is related to how subjects used their mobile phone in public places.

There are some methodological limitations to this study. Recruitment method limits the validity of the study. With the method designed as an online survey, possible self-selection processes should be noted. Online surveys are predisposed to an inherent selection bias. It can be hypothesized that smartphone users in particular found it appealing to participate in the survey, who are trying to relativize the negative image of smartphone dependency. As a further limitation, subjects were mainly psychology/psychotherapy students, and as a consequence, age distribution was very narrow. Female participants contributed disproportionately to the respondent data set. However, this gender bias in online surveys has been frequently observed in the literature (44). Further studies with a broader recruitment method are needed to generate more representative data and confirm discussed results.

In conclusion, results emphasize the importance of attachment-based therapeutic intervention techniques in addiction therapy (45–47). Psychotherapeutic interventions aiming at the attachment style can be helpful in dealing with emotional stress and thereby prevent using the smartphone dysfunctionally to influence emotions. Perhaps the most important and hitherto most widely accepted therapeutic implication of attachment theory is the reference to the importance of the therapeutic relationship. The importance of the therapeutic relationship at the beginning of an addiction therapy for the further course of a treatment has been shown in a plethora of research (32). At best, this can become a corrective relationship experience that leads to a more secure attachment style.

## Data Availability

The datasets generated for this study are available on request to the corresponding author.

## Ethics Statement

The studies involving human participants were reviewed and approved by Ethics Commission of the Faculty of Psychotherapy Science and the Faculty of Psychology. The patients/participants provided their written informed consent to participate in this study.

## Author Contributions

CE was involved in planning and supervising the work. AS processed the experimental data, performed the analysis, and drafted the manuscript. MS took the lead in writing the manuscript. All authors provided critical feedback and helped shape the research, analysis, and manuscript.

## Funding

This work was supported by the German Research Foundation (DFG) and the Technical University of Munich (TUM) in the framework of the Open Access Publishing Program.

## Conflict of Interest Statement

The authors declare that the research was conducted in the absence of any commercial or financial relationships that could be construed as a potential conflict of interest.
